# Gut development following insulin-like growth factor-1 supplementation to preterm pigs

**DOI:** 10.1038/s41390-023-02949-9

**Published:** 2023-12-12

**Authors:** Martin Bo Rasmussen, Kristine Holgersen, Stanislava Pankratova, Ole Bæk, Douglas G. Burrin, Thomas Thymann, Per Torp Sangild

**Affiliations:** 1 Comparative Pediatrics and Nutrition, Department of Veterinary and Animal Sciences, Faculty of Health and Medical Sciences, University of Copenhagen, Frederiksberg, Denmark; 2 Hans Christian Andersen Children’s Hospital, Odense University Hospital, Odense, Denmark; 3 US Department of Agriculture/Agricultural Research Service and Department of Pediatrics, Baylor College of Medicine/Texas Children’s Hospital, Children’s Nutrition Research Center, Houston, TX USA; 4 Faculty of Theology, University of Copenhagen, Copenhagen, Denmark; 5 Department of Neonatology, Rigshospitalet, Copenhagen, Denmark

## Abstract

**Background:**

Reduced insulin-like growth factor-1 (IGF-1) levels may contribute to impaired organ development in preterm infants. Using preterm pigs as a model, we hypothesized that IGF-1 supplementation improves health and gut development during the first three weeks of life.

**Methods:**

First, clinical and organ endpoints were compared between artificially-reared, cesarean-delivered preterm pigs and vaginally-delivered, sow-reared term pigs at 5, 9 and 19 days. Next, preterm pigs were treated with recombinant human IGF-1 for 19 days (2.25 mg/kg/day, systemically).

**Results:**

Relative to term pigs, preterm pigs had lower body weight, fat, bone contents, relative weights of liver and spleen and a longer and thinner intestine at 19 days. Preterm birth reduced intestinal villi heights and peptidase activities, but only at 5 and 9 days. In preterm pigs, IGF-1 reduced mortality primarily occurring from gastrointestinal complications and with a tendency towards salvaging smaller pigs. IGF-1 supplementation also increased spleen and kidney weights, small intestine length and maltase to lactase activity, reflecting gut maturation.

**Conclusion:**

Preterm birth affects body composition and gut maturation in the first 1–2 weeks, but differences are marginal thereafter. Supplemental IGF-1 may improve gut health in pigs and infants in the first few weeks after preterm birth.

**Impact:**

- Insulin-like growth factor 1 (IGF-1) supplementation may improve gut health and development in prematurity, but whether the effects are sustained beyond the immediate postnatal period is unclear.
- In preterm pigs, the prematurity effects on IGF-1 and gut health deficiencies are most pronounced during the first week of life and diminishes thereafter.
- In preterm pigs, IGF-1 supplementation beyond the first week of life reduced mortality.
- The present study provides evidence of a sustained effect of IGF-1 supplementation on the gastrointestinal tract after the immediate postnatal period.

## Introduction

Preterm birth represents ~10% of all live births and increases the risk of immaturity-related morbidities such as bronchopulmonary dysplasia and necrotizing enterocolitis (NEC),^1–4^ potentially leading to impaired growth ^5,6^ and neurodevelopment^7,8^ These conditions relate to nutritional and endocrine deficits stemming from premature dissociation from the maternal-placental-fetal unit.^9^ Nutrient and energy deficits may result from accelerated organ development, impaired nutrient absorption and enteral feeding intolerance.^10^ Endocrine deficiencies include disturbances in the levels and effects of important regulators of growth and organ development, such as insulin and insulin-like growth factor-1 (IGF-1). Despite advancements in nutritional strategies, strategies to correct endocrine deficits have received limited attention due to knowledge gaps and concerns about replacement therapies’ safety and efficacy.

IGF-1, produced primarily in hepatocytes but also many other cell types,^11–13^ is crucial in cell growth and normal postnatal development in mammals. Preterm infants experience a prolonged period of reduced circulating IGF-1 levels compared with term infants and age-matched fetuses *in-utero*.^11,14^ Reduced IGF-1 levels are associated with dysfunctional lungs,^15^ brain,^16,17^ eyes,^18^ and gut^19^ but cause-effect relationships are unclear. In a randomized placebo-controlled trial, 121 extremely premature infants were allocated to supplementation with recombinant human (rh)IGF-1 or placebo.^20^ While IGF-1 supplementation did not affect the primary outcome (retinopathy of prematurity), it was associated with a reduced incidence of bronchopulmonary dysplasia and intraventricular hemorrhage. IGF-1 also affects bone growth *by regulating* chondrocyte, osteoblast and osteocyte differentiation, maturation and function.^21^ Thus, circulating IGF-1 levels are positively associated with anthropometric growth indices at birth^22–24^ and postnatally^19,25–27^ in very preterm infants. This is supported by in vivo studies demonstrating that IGF-1 deficient mice show delayed growth^28^ and reduced bone density, volume and thickness.^29^

In the gut, supplemental IGF-1 promotes intestinal epithelium cell proliferation and differentiation, improves gut barrier function, and reduces apoptosis.^30–36^ Preterm pigs exhibit postnatal circulating IGF-1 deficiency and IGF-1 supplementation for 4–8 days improves gut maturation.^37–39^ However, the impact of IGF-1 supplementation beyond this period remains uncertain. In pigs, some effects of preterm birth on gut structure and function resolve within the first weeks of life but certain deficits may persist.^40^ For instance, preterm pigs have lower brush-border sucrase and maltase activities four weeks after birth when rearing conditions are identical. Conversely, lactase and peptidase activities become more similar within the first weeks.^40^ Additionally, cesarean section and artificial rearing, irrespective of gestational age at birth, can independently affect gut functions and circulating IGF-1 levels.^37,41^ To fully understand the consequence of preterm birth, it is essential to understand both the effects of reduced gestational age at birth and the effects of the special delivery and clinical care procedures associated with preterm birth, like incubators-rearing, limited maternal contact, specialized care and parenteral/enteral nutrition.

To investigate the combined effect of ontogenetic immaturity and artificial rearing on clinical adaptation and organ development, we first compared preterm, cesarean-delivered, artificially-reared piglets with groups of term, vaginally-delivered pigs reared by their sow. We compared organ dimensions and development of gastrointestinal digestive enzymes and morphology at 5, 9 and 19 postnatal days. Next, we randomized preterm pigs to IGF-1 supplementation or placebo for 19 days. We hypothesized that restoring circulating IGF-1 levels to normo-physiological levels would alleviate the detrimental effects of preterm birth on clinical variables and postnatal organ development beyond the immediate neonatal period, with a focus on gut growth, digestive enzyme activities and gastrointestinal morphology. Our studies are important to help understand the potential gut effects of IGF-1 supplementation of extremely preterm infants, currently being tested in a large international multicenter trial (Clinical Trials Registry: NCT03253263).

## Materials and methods

### Animals and experimental setup

Danish Animal Experiments Inspectorate approved the study and it was conducted in accordance with the European Communities Council Directive 2010/63/EU. Procedures aimed to follow ARRIVE guidelines for animal experimentation^42^ and all personnel participating in the study were blinded to interventions.

**Fig. 1:**
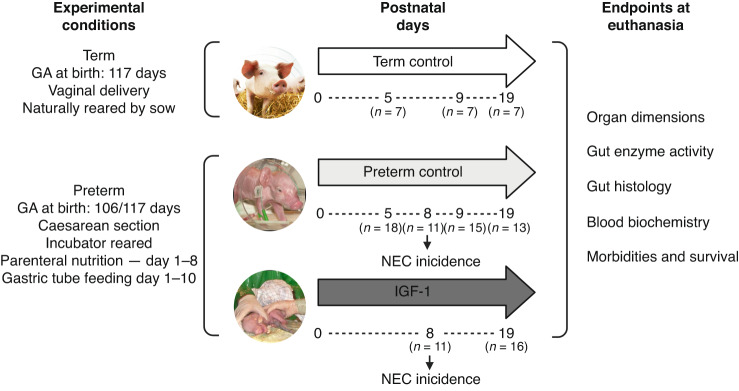
Overview of the study design. Organ dimensions, gastrointestinal enzymes and histology were compared between naturally-reared term-born pigs and preterm pigs born at 90% of full gestation at postnatal day 5 (*n* = 7 vs *n* = 18), 9 (*n* = 7 vs *n* = 15) and 19 (*n* = 7 vs *n* = 13). Preterm control pigs reared for 19 days were compared with preterm pigs treated with recombinant human IGF-1 (2.25 mg/kg/day). Preterm pigs were fed via the gastric tube until postnatal day 10 while parental nutrition was gradually decreased and stopped by day 8. At day 8, the incidence of necrotizing enterocolitis was compared in a subset of preterm control and IGF-1 pigs (both, *n* = 11), while remaining measured variables were compared in preterm controls and IGF-1 pigs at day 19 (*n* = 13 vs *n* = 16). IGF-1 insulin-like growth factor-1, GA gestational age at birth, NEC necrotizing enterocolitis.

Figure 1 describes the experimental design. Term pigs (Landrace × Yorkshire × Duroc) were born vaginally at full term (117 ± 2 days) and reared by their sow at farm conditions and brought for euthanasia and tissue collection in the morning of postnatal day (PND) 5, 9 or 19 (*n* = 7 for each time point). Further, tissues were collected and analyzed from control (cesarean-derived, incubator-reared) preterm pigs at PND5 (*n* = 18) and PND9 (*n* = 15), as described in previous studies.^39^

For the 19 day study on IGF-1 supplementation, 66 preterm pigs were delivered by cesarean section in three sows at 90% of gestation (106 days), resuscitated and housed in experimental incubators, as described previously.^37,43^ The sample size of preterm groups was based on previous IGF-1 supplementation studies with moderate gut effects.^39^ One pig did not recover from resuscitation and was euthanized before randomization. After stabilization, the pigs were fitted with orogastric tubes and umbilical artery catheters and block-randomized according to birth weight and sex receiving either IGF-1 supplementation with a 1:1 molar ratio of rhIGF-1/binding protein-3 (BP-3) (mecasermin rinfabate, *n* = 32) or equivalent amounts of vehicle buffer (50 nM sodium acetate, 105 mM sodium chloride, 0.005% (v/v) polysorbate 20, pH 5.5, Takeda, Cambridge, MA, *n* = 33). We aimed for circulating IGF-1 levels of 30–110 ng/mL, based on previous studies in infants and piglets.^37^ A dose of 2.25 mg/kg/day rhIGF-1/BP-3 (10 mg/mL, ~0.225 mL/kg/day) or an equal volume of vehicle was administered continuously via parental nutrition at days 1–7 (intra-arterially, i.a.). After catheter withdrawal on PND8, supplemental IGF-1 was administered three times daily *via* a subcutaneous catheter (Unomedical, Lejre, Denmark) with doses of 0.75 mg/kg (2 mg/mL, ~0.38 mL/kg). To account for an increased metabolic rate in smaller animals, IGF-1/vehicle dosing was adjusted to metabolic body weight (multiplication of dose with body weight^0.7^/body weight). The pigs were passively immunized with sows’ plasma during the first 24 hours of life (20 mL/kg). All animals were treated with prophylactic oral antibiotics (amoxicillin with clavulanic acid, 2care4, Esbjerg, Denmark), gentamicin (Gentocin, Scanvet, Fredensborg, Denmark) combined with metronidazole (Flagyl, Sanofi, Paris, France) at PND8-10 to prevent diarrhea. On PND11, the pigs were transferred to larger cages with free access to drinking water and in-cage enrichments. Preterm pigs from two litters (*n* = 43) were reared for 19 days. The remaining pigs (*n* = 11 in each group) were euthanized on PND8 to assess the incidence of NEC lesions. The investigators were blinded to the intervention groups.

### Feeding and nutrition

The preterm pigs received continuous parental nutrition (PN, Kabiven, Fresenius Kabi, Uppsala, Sweden) *via* the umbilical catheter at PND1-7, with infusion rates of 3–6 mL/kg/h. The PN was modified with Vamin, Soluvit, Vitalipid, and Peditrace (all Fresenius Kabi). Enteral nutrition (EN) gradually increased (32–224 mL/kg/day) throughout the study. Pigs were fed through the orogastric tube until PND5 and drank independently from a trough from PND6-10. The EN consisted of bovine milk supplemented with vitamins and minerals, a diet that minimizes gut complications in long-term preterm pig studies.^44^ The PN and EN supplies were adjusted for metabolic body weight of pigs (Supplementary Table S1).

### Clinical evaluation, tissue collection and NEC evaluation

Health status was evaluated from weight gain and clinical/fecal scorings twice daily, using validated scoring systems.^38^ According to pre-defined human endpoints, pigs were euthanized if signs of respiratory distress, circulatory compromise, severe lethargy or severe pain were observed. Clinical score and organ autopsy were used to determine the cause of early euthanasia. All three litters were included in the survival analysis, excluding pigs that died from iatrogenic causes, i.e., umbilical catheter displacement leading to bleeding, intra-abdominal infusion of parenteral nutrition or occlusion of hind leg arterial perfusion, causing irreversible leg hypoperfusion and pain. At PND19, pigs were subjected to whole-body dual-energy X-ray absorptiometry (DEXA), as previously described.^38^ The pigs were sacrificed with intracardial sodium-pentobarbital (Euthanimal, ScanVet, Animal Health, Denmark). All pigs were fed 25 mL/kg bovine milk three hours before sacrifice. The organs were weighed and gut tissue samples were snap-frozen at -80 °C or fixed in 4% formaldehyde (CellPath, Newtown, Powys, United Kingdom) for analyses of enzyme activity and villous morphology.

Macroscopic NEC scoring was done by two blinded independent observers, assessing the proximal, middle and distal parts of the small intestine (SI) as well as the stomach and colon, using the following scoring system: 1 = absence of lesions, 2 = local hyperemia, 3 = hyperemia, extensive edema and local hemorrhage, 4 = extensive hemorrhage, 5 = local necrosis or pneumatosis intestinalis, 6 = extensive necrosis and pneumatosis intestinalis. A score of 4 in any gastrointestinal region was defined as a case of ‘NEC’.

### Circulating IGF-1 levels, plasma biochemistry, blood glucose, and insulin

At PND19, blood was collected and stored at -80 °C until analysis. Biochemistry was analyzed in lithium-heparinized plasma with an Advia 1800 Chemistry system (Siemens, Ballerup, Denmark). Glucose and insulin levels were analyzed using the glucose-oxidase method (model 2300; Yellow Spring Instruments, Yellow Spring, OH) or radioimmunoassay (EMD Millipore Corporation, Billerica, MA), respectively. Fasting glucose levels measured at PND4, PND8, and PND12 with a glucometer (ACCU-CHEK, Roche Diagnostics, Hvidovre, Denmark). Circulating IGF-1 levels were measured in one litter (IGF-1: *n* = 9, control: *n* = 8) at PND18, 60 min after subcutaneous rhIGF-1/vehicle injection. IGF-1 levels were quantified with a human IGF-1 ELISA kit (Mediagnost GmbH, Reutlingen, Germany). The limit of quantification was 20 ng/mL and values below were assigned a value of 10 ng/mL for quantitative evaluations. rhIGF-1/IGFBP3 autoantibodies were detected in 1 × 100 diluted plasma samples collected on day 19 using a porcine-specific ELISA kit (Genemed Synthesis, San Antonio, Texas).

### Gut morphology and enzyme activity

The SI proximal region at PND5, -9, and -19 and the distal region at PND19 were stained with hematoxylin and eosin, as previously described.^37^ Images were captured with a Leica 2500 optical microscope and villus height and crypt depth were quantified as an average of 10 representative regions measured with ImageJ software (Laboratory for Optical and Computational Instrumentation, University of Wisconsin-Madison). Brush border enzyme activities (hydrolytic units, U) were assessed in the proximal and distal SI regions as previously described.^45^ The total SI hydrolytic capacity from each brush border enzyme was calculated as the mean enzymatic activity (U/g) from proximal and distal SI regions multiplied by total SI weight per kg bodyweight.

### Statistics

Assumptions of normality were tested using the Shapiro-Wilk test. Homoscedasticity was assessed by visual inspection of residual plots. If assumptions were violated, non-parametric analysis was performed. Binary outcomes were analyzed by logistic regression. Continuous variables were analyzed using a generalized linear model and repeated measures were analyzed using a generalized linear mixed model with the individual pig as a random factor. Survival was analyzed using Cox proportional-hazards model. Models were adjusted for sex, litter, and birth weight as appropriate. Tukey’s honest significance test or Dunn’s test with Benjamini-Hochberg adjustment was used to correct for multiple comparisons. A *p*- value below 0.05 was considered statistically significant. Values are given as means ± SEM unless otherwise specified.

## Results

### Comparison of preterm and term pigs

#### IGF-1 levels, organ growth and body composition

Circulating levels of IGF-1 did not differ between preterm and term pigs at PND5 (23 ± 2 vs. 16 ± 3 ng/ml) but were lower in preterm pigs at PND9 (19 ± 3 vs. 47 ± 7 ng/ml, *p* = 0.004) and more similar again at PND19 (34 ± 6 vs. 46 ± 9 ng/ml).

**Fig. 2:**
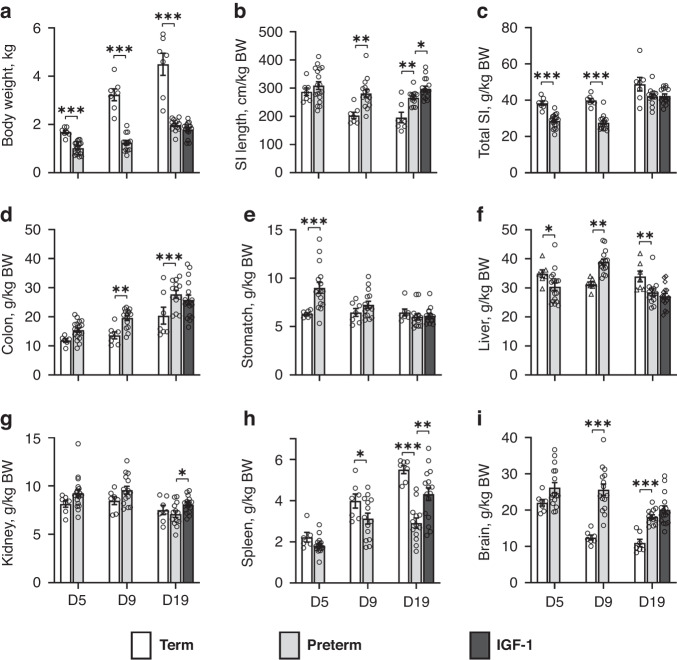
Body weight and organ dimensions in term, preterm control and preterm IGF-1 pigs. Body weight and organ growth (g/kg body weight, **a**–**i**) in term (all *n* = 7) and preterm pigs at postnatal ages 5 days (*n* = 18), 9 days (*n* = 15) and 19 days (*n* = 13), and in preterm pigs supplemented with IGF-1 until 19 days (IGF-1, *n* = 16). BW body weight, SI small intestine. Values are means ± SEM. *p < 0.05, **p < 0.01, ***p < 0.001.

Compared with term pigs, body weight was lower in preterm pigs at PND5, -9, and -19 (all *p* < 0.01, Fig. 2A). Regarding preterm-term organ dimensions, preterm pigs had longer SI than term pigs at PND9 and -19 (Fig. 2B, *p* < 0.001), but reduced relative intestinal weight, compared with term pigs (*p* < 0.05 at PND5 and -9, Fig. 2C). Thus, preterm pigs seem to have a long and light intestine. Contrary to SI weights, preterm pigs showed a relative increase in colon weights, compared with term pigs (*p* < 0.01 at PND9 and -19, Fig. 2D), while stomach weights were elevated only in the first week (*p* < 0.01 on PND5, Fig. 2E). Preterm pigs showed increased relative liver weight at PND9 (*p* < 0.01) and decreased weight at PND5 and- 19 (*p* < 0.05, Fig. 2F), compared with term controls. The relative spleen weights increased markedly with postnatal age, but less so in preterm pigs (decreased weights at PND9 and -19, *p* < 0.05, Fig. 2H). Conversely, the relative brain weights decreased with advancing postnatal age but less in preterm than in term pigs (*p* < 0.001 for PND9 and -19, Fig. 2I).

At PND19, preterm pigs had lower body fat percentage (3.64 ± 0.26%, *n* = 10 *vs*. 9.49 ± 0.75%, *n* = 7, *p* < 0.001) and bone mineral density (BMD) (0.20 ± 0.01 *vs*. 0.26 ± 0.01 g/cm^2^, *p* < 0.001) than term pigs. There were no preterm-term differences in the proportion of bone in the body (1.64 ± 0.04 vs. 1.67 ± 0.04%).

#### Blood biochemistry, gut digestive enzyme activities and mucosal morphology

At PND19, preterm pigs showed reduced levels of albumin, total protein, alanine aminotransferase, cholesterol, creatinine, blood urea nitrogen, iron, calcium, phosphate, glucose and insulin, while basic phosphatase, gamma-glutamyltransferase, and magnesium were increased, compared with term pigs (Table 1).

**Table 1:** Biochemistry results among term and preterm pigs supplemented with rhIGF-1 or vehicle for 19 days.

	Term	Preterm	IGF-1	*P*_Term *vs*. Preterm_	*P*_rhIGF-1 *vs*. Preterm_
Number of animals	7	13	16		
Albumin g/L	29.5 ± 1.5	18.3 ± 0.6	17.0 ± 0.9	<0.001	0.14
Total protein g/L	47.5 ± 2.1	31.2 ± 0.7	30.0 ± 1.3	<0.001	0.17
Alkaline/basic phosphatase, U/L	834.9 ± 83.9	1439.9 ± 152.3	1279 ± 102	0.003	0.14
Alanine aminotransferase, U/L	41.9 ± 3.3	31.8 ± 1.6	32.6 ± 1.5	0.005	0.77
Aspartate aminotransferase, U/L	56.7 ± 15.0	41.2 ± 9.6	32.8 ± 2.8	0.138	0.61
Gamma-glutamyltransferase, U/L	10.0 ± 2.5	21.9 ± 2.8	18.6 ± 2.3	0.005	0.34
Bilirubin, µmol/L	1.9 ± 0.4	1.5 ± 0.2	1.4 ± 0.2	0.516	0.16
Cholesterol, mmol/L	3.8 ± 0.4	2.8 ± 0.1	2.5 ± 0.1	0.006	0.07
Creatine Kinase, U/L	488.0 ± 208.4	387 ± 95	252 ± 30	0.492	0.68
Creatinine, µmol/L	66.7 ± 7.2	47.5 ± 2.6	40.4 ± 1.4	0.007	0.04
Blood urea nitrogen, mmol/L	3.0 ± 0.2	1.8 ± 0.3	1.9 ± 0.3	0.012	0.90
Iron, µmol/L	19.5 ± 2.7	4.6 ± 1.7	3.1 ± 1.4	<0.001	0.14
Calcium, mmol/L	3.0 ± 0.1	2.6 ± 0.1	2.5 ± 0.1	0.009	0.30
Inositol phosphate, mmol/L	3.0 ± 0.2	2.7 ± 0.1	2.4 ± 0.1	0.016	0.02
Magnesium, mmol/L	1.0 ± 0.1	1.3 ± 0.1	1.1 ± 0.1	0.018	0.05
Sodium, mmol/L	143.1 ± 3.2	143.1 ± 2.0	144.1 ± 2.2	0.926	0.81
Potassium, mmol/L	4.9 ± 0.5	4.5 ± 0.1	4.2 ± 0.1	0.254	0.07
Glucose, mmol/L	19.0 ± 1.6	11.0 ± 1.1	10.5 ± 0.9	<0.001	0.69
Insulin, mU/L	2.4 ± 0.3	0.4 ± 0.2	0.8 ± 0.3	0.002	0.22

Blood samples were collected prior to euthanasia at day 19. Values are means ± SEM.

**Fig. 3:**
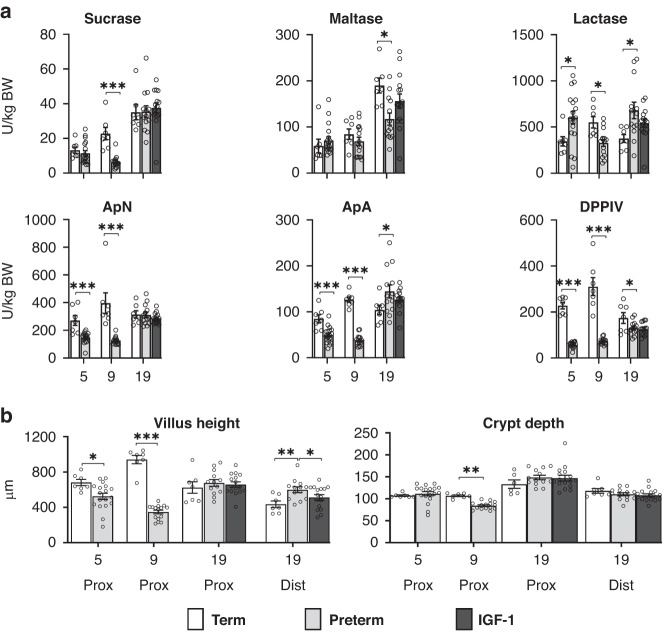
Gastrointestinal enzyme activity, villus height and crypt depth among term, preterm control and preterm IGF-1 pigs. Total small intestinal hydrolytic capacity (units/kg bodyweight) from each brush border enzyme (**a**) and small intestinal histology (**b**) in term (all *n* = 7) and preterm pigs at postnatal ages 5 days (*n* = 18), 9 days (*n* = 15) and 19 days (*n* = 13), and in preterm pigs supplemented with rhIGF-1 until 19 days (IGF-1, *n* = 16). ApN aminopeptidase N, ApA aminopeptidase A, DPPIV dipeptidylpeptidase, Prox proximal intestine, Dist distal intestine, BW body weight. Values are means ± SEM. *p < 0.05, **p < 0.01, ***p < 0.001.

The total small intestinal enzymatic hydrolytic capacity relative to body weight was reduced in preterm *vs*. term pigs for all peptidases (ApN, ApA and DPPIV) at PND5-9 (all *p* < 0.01) and for sucrase and lactase at PND9 (Fig. 3A), while lactase was increased in preterm pigs at PND5. At PND19, less consistent preterm-term differences were evident for the enzyme activities, but lactase was higher and maltase lower in the preterm pigs (both *p* < 0.05), resulting in a markedly reduced maltase-to-lactase ratio in preterm pigs at this time (0.19 ± 0.02 *vs*. 0.55 ± 0.08, *p* < 0.001), reflecting delayed brush-border enzyme maturation.

For tissue-specific hydrolytic enzyme activity (U/g of tissue, Supplementary Fig. 1A–C), preterm pigs had decreased sucrase activity in the proximal SI at PND5 to -9 and maltase activity in the distal SI at PND19 (all *p* < 0.05). Conversely, lactase activity was increased in the proximal and distal SI at PND5 and in the distal SI at PND19 in preterm pigs, while distal SI maltase activity was increased at PND5 and -9. For peptidases, preterm pigs had reduced ApN activity at PND5 (only distal region) and PND9 (both regions), reduced ApA activity at PND9 (both regions), and reduced DPPIV activity at PND5 (both regions) and PND9 (only distal region, all *p* < 0.05, Supplementary Fig. 1D–F). In contrast, proximal SI ApN and ApA activities were increased in preterm pigs at PND19.

Preterm pigs had shorter villi at PND5 and -9 (*p* < 0.05) and shorter crypts at PND9 (*p* < 0.05) than term pigs (proximal SI, Fig. 3B). At PND19, distal villi were higher in preterm *vs*. term pigs (*p* < 0.01, indicating less advanced maturation).

### IGF-1 supplementation until day 19 in preterm pigs

#### Circulating IGF-1 levels, morbidities and mortality

IGF-1 pigs had higher IGF-1 levels than control pigs 60 min after rhIGF-1/vehicle injection at PND18 (139 ± 10 *vs*. 43 ± 3 ng/ml, *p* < 0.001). rhIGF1 autoantibodies were higher in preterm IGF-1 pigs compared with controls at PND19 (*p* = <0.01, Supplementary Fig. 2). Nine IGF-1 pigs and 17 control pigs died or were euthanized ahead of the predetermined end of the experiment due to respiratory distress (3/26: one IGF-1, two controls), clinical gastrointestinal symptoms with feeding intolerance and/or signs of NEC (18/26: six IGF-1, 12 controls), unknown cause (1/26, one control) and catheter-related problems (two IGF-1, two controls). A total of 24 of the 26 early deaths occurred before PND11. Details of the timing and cause of death are shown in Supplementary Table S2. The remaining preterm pigs were sacrificed as planned, either on PND8 (seven IGF-1, three controls) or PND19 (16 IGF-1, 13 controls). When the four pigs with catheter-related problems were excluded from the survival analysis, IGF-1 pigs showed reduced mortality before the predefined time of euthanasia (7/30 *vs*. 15/31, Hazard ratio: 0.40 (CI: 0.16;0.98), *p* = 0.046). During the first postnatal week (when preterm pigs display the highest NEC sensitivity), the incidence of NEC, as detected in one litter, was similar among IGF-1 and control pigs (5/11 vs. 7/11, *p* = 0.43). No pigs had NEC lesions at autopsy on PND19.

#### Growth, organ development and body composition

The IGF-1 pigs that survived until PND19 tended to have lower mean birth weight (903 ± 48 vs. 996 ± 50 g, *p* = 0.15) and weight at PND19 (1793 ± 76 vs. 1976 ± 70 g, *p* = 0.09) than controls. Daily weight gain was unaffected by IGF-1 supplementation (26 ± 1 *vs*. 26 ± 1 g/kg/day). At PND19, IGF-1 pigs had increased SI length (*p* < 0.05, Fig. 2B), kidney weight (*p* < 0.05, Fig. 2G) and spleen weight (*p* < 0.01, Fig. 2H) compared with control pigs. Relative SI weight (i.e. weight per kg body weight) was similar between groups. IGF-1 pigs had reduced BMD (0.199 ± 0.005 g/cm^2^, *n* = 10) compared with controls (0.188 ± 0.004 g/cm^2^, *n* = 15, *p* < 0.05), but similar fat and bone percentage. When adjusting for body weight at PND19, the difference in BMD was no longer statistically significant (*p* = 0.13).

#### Blood biochemistry, gut digestive enzyme activities and mucosal morphology

At PND19, IGF-1 pigs had reduced phosphate and creatinine levels compared with control pigs (Table 1). Blood glucose values, as measured by a glucometer, were similar between IGF-1 and control pigs, both at PND4 (4.6 ± 0.3 vs. 4.9 ± 0.2 mmol/L, *n* = 19–20) and PND8 (3.3 ± 0.2 *vs*. 3.7 ± 0.2 mmol/L, *n* = 17–18). By 12 days of age, glucose values were lowered in IGF-1 pigs (3.2 ± 0.3 vs. 3.8 ± 0.2 mmol/L, *n* = 14-17, *p* < 0.05) but at PND19, no differences were seen in glucose levels, blood biochemistry or insulin levels.

There was no significant effect of IGF-1 supplementation on disaccharidase and peptidase total small intestinal enzymatic hydrolytic capacity except a tendency to reduce lactase activity (*p* = 0.07, Fig. 3A). However, the maltase-to-lactase ratio was higher in IGF-1 pigs relative to controls (0.31 ± 0.03 vs. 0.19 ± 0.02, *p* < 0.05) as a sign of improved gastrointestinal maturation. For tissue-specific enzyme activities, IGF-1 supplementation reduced distal SI lactase activities (*p* < 0.05, Supplementary Fig. 1C). The IGF-1 supplementation exerted limited effects on gut morphology (e.g. reduced distal villi height, *p* < 0.05, Fig. 3B).

## Discussion

IGF-1 supplementation may alleviate the multi-organ effects of preterm birth.^11^ Still, the effects on gut development from currently available infant or animal studies are poorly described. To understand prematurity-related gastrointestinal and organ growth deficits, we first compared artificially-reared preterm pigs with naturally-reared farm pigs. Preterm pigs showed deficient body growth and altered growth trajectories for SI, colon, liver, spleen and brain compared with term pigs, together with immature intestinal digestive enzymes (e.g., reduced peptidase activities in the first weeks, reduced maltase/lactase activity at 19 days). IGF-1 supplementation in preterm pigs improved overall viability, primarily by reducing mortality due to clinical gastrointestinal symptoms, increased SI length and had marginal gut enzyme maturation effects. The exact mechanism behind the increased viability remains unclear but confirms observations from another preterm pig study with eight days IGF-1 supplementation.^39^

Only two pigs, one from each group, died after PND11, indicating that IGF-1’s clinical effects occurred in the immediate postnatal period. In extremely preterm infants, more than 50% of deaths also occur during the first 10 days of life^46,47^ and preterm neonates may be most deficient in intestinal IGF-1 during the immediate postnatal period before endogenous IGF-1 production increases. Consequently, the IGF-1 effects on neonatal viability in preterm pigs fitted the temporal pattern of IGF-1 deficiency and mortality in preterm infants. Accordingly, we observed the biggest gap between plasma IGF-1 levels in preterm and term animals at PND9. This effect may arise from improved gut function, as indicated by the tendency of IGF-1 supplementation to reduce NEC lesions in preterm pigs and newborn rodents.^37,39,48^ Supplemental IGF-1 tended to support survival in preterm pigs with lower weight. Such increased survival among smaller/weaker pigs in the IGF-1 group could bias towards more detrimental outcomes, potentially leading to underestimation of the beneficial maturational effects of IGF-1 supplementations seen in our study. However, in our previous study assessing brain outcomes after IGF-1 supplementation, most findings were unchanged after excluding low birth weight pigs in a sensitivity analysis.^49^ The limited sample size prevented in-depth analyses of the sub-group of small piglets in the current study.

Organ weights and changes across the different postnatal ages among preterm pigs were consistent with our previous findings.^43^ Preterm pigs had a ‘long and slender’ SI with reduced weight and increased length relative to term pigs. However, when preterm and term pigs were born and reared identically, the intestinal weight deficits were less pronounced.^38,41,50^ These findings emphasize the impact of birth method, rearing environment and diet on gut maturation in addition to gestational age at birth. The brain was less affected by body growth rate and environment (“brain sparring”), explaining the increased relative brain weights in preterm pigs compared to term pigs. Consistent with a previous study,^41^ the preterm-term differences in plasma biochemistry may reflect the slower growth and altered metabolism in preterm animals (e.g., reduced creatinine), immature liver (e.g., reduced albumin) and dysfunctional endocrine pancreas (e.g., reduced insulin).

IGF-1 supplementation increased kidney and spleen weights but other organ weights remained unaffected, despite the fact that IGF-1 stimulates protein synthesis in the small intestine and the brain of preterm pigs.^39,51^ Brain outcomes have been reported elsewhere.^49^ Preterm pigs show increased (rather than reduced) fat mass,^38^ indicating an interaction between the effect of preterm birth, nutrient intake and growth rate on body composition as seen in infants.^52,53^ Likewise, the observed reduced BMD in preterm pigs may result from poor nutrient intake and growth rates, although separate effects of preterm birth itself cannot be excluded.^43^ The marginal decrease in BMD in the IGF-1 pigs conflicts with other evidence suggesting that IGF-1 promotes bone formation and growth.^54^ However, this effect may be partly explained by the fact that more of the weaker/smaller pigs survived in the IGF-1 group and post-hoc analysis adjusting for body weight abolished the effect of IGF-1 supplementation on bone mineralization.

IGF-1 supplementation for 19 days showed limited impact on tissue-specific intestinal enzyme activities and total hydrolytic capacity. However, the increased maltase-lactase ratio suggests effects on epithelial maturation, possibly related to IGF-1’s influence on intestinal epithelial stem cells at the crypt basis.^55^ Considering the effects on intestinal growth and enzyme activities in our previous short-term studies,^37,39^ the limited effects of 19 days of supplementation were surprising but may be explained by the occurrence of systemic rhIGF-1/BP3 autoantibodies at PND19.

Circulating IGF-1 levels and intestinal villus/crypt dimensions appeared mostly affected by preterm birth on day 5 and 9. Enzyme activities were generally lower in preterm pigs at this time for sucrase, ApN, ApA and DPPIV. However, lactase and maltase activities tended to increase. Consistent with this, lactase activity largely depends on postconceptional age, while sucrase and maltase activities depend on combinations of postnatal age, environment and diet.^50^ In previous studies, preterm pigs had lower disaccharidase activities than identically reared term pigs,^40^ while naturally-reared term pigs had higher digestive enzyme activities than artificially-reared term pigs.^41^ Further, prematurity-related intestinal weight reduction inevitably results in a reduction in the estimated total digestive capacity. Thus, in this study, preterm pigs had lower total enzymatic sucrase activity in the first postnatal weeks, while lactase activity was increased compared to term pigs. The resulting reduction in maltase-lactase ratio suggests a persistent delay in postnatal gut maturation 19 days in preterm pigs.^56^ Peptidase activities were initially reduced in preterm pigs. However, the effects disappeared after 19 days, suggesting that the postnatal factors resolve the initial effect of preterm birth on peptidases, as previously described.^40^

IGF-1 can bind to the insulin receptor with reduced affinity compared to insulin^11^, promoting insulin sensitivity in muscles.^57^ In our previous studies, IGF-1 supplementation failed to affect the hyperglycemia during the first week of life and did not induce hypoglycemia.^37,39^ In this study, preterm pigs showed reduced glucose and insulin levels at PND19. While IGF-1 supplementation marginally reduced blood glucose after 12 days (but not at 4, 8 or 19 days), insulin levels were unaffected. Glucose values remained within normal range, consistent with no signs of hypoglycemia in a recent clinical IGF-1 trial.^20^

IGF-1 supplementation increased spleen weights, but its effects on hematology and systemic immunity variables have remained sparse.^37,58^ Adult mice treated with IGF-1 for 7-14 days had increased spleen, thymus and kidney weights.^59^ The increased spleen weight was attributed to elevated T-cell and B-cell numbers, while only neutrophil counts were increased in the peripheral blood. As seen in our study, IGF-1 deficits due to preterm birth may also affect kidney maturation, which is consistent with observations in IGF-1 deficient mice.^60^ IGF-1 may stimulate the fluid-retaining properties through tubular reabsorption,^61^ but we did not observe any differences in kidney-related biochemistry.

The present study has several limitations and caution is needed when translating findings from preterm pigs to preterm infants. Preterm pigs born at 90% of full gestation exhibit highly immature gut functions, with the highest NEC-sensitivity and mortality in the first 1–2 weeks of life,^43,62^ while extremely preterm infants remain sensitive for a longer period.^63^ Differences in interspecies organ development trajectories warrant careful consideration when applying results of experimental IGF-1 therapy studies to humans, particularly given its multi-organ effects. Our sample size estimations were pragmatic and based on experience from previous studies of gut development after IGF-1 supplementation. Considering the multiple endpoints recorded and their observed variation, it is acknowledged that greater sample sizes (especially for the term reference group) could have strengthened conclusions. The improved clinical condition of IGF-1 supplemented pigs was important and our blinded study minimizes the risk of bias in the decisions to euthanize due to poor clinical condition. However, due to limited sample size, our results on the IGF-1 effect on viability must be interpreted with caution. While we aimed to study preterm birth and IGF-1 effects beyond the immediate postnatal period, 19 days in pigs may be insufficient to fully capture catch-up growth and reflect the long period of hospitalization for very preterm infants. Finally, it is important to note that the observed preterm-term differences reflect the combined effect of reduced gestational age at birth and environmental factors like delivery mode, artificial versus natural rearing and maternal care.

In conclusion, preterm pigs show altered body composition, organ growth and gut structure and function relative to term, naturally-reared animals. These differences are most pronounced in the first 1–2 weeks after preterm birth. By three weeks of age, IGF-1 supplementation to preterm pigs moderately increases kidney and spleen weights, has marginal gut maturational effects and increases overall survival, primarily by reducing gastrointestinal symptoms. The mechanisms of IGF-1 effects following preterm birth remain to be better understood and more knowledge is important for future decisions to implement IGF-1 replacement therapy for very preterm infants in the first weeks after birth.

### Supplementary information

Supplementary Information

## Data Availability

The datasets generated during and/or analyzed during the current study are available from the corresponding author upon request.
