# Supplementary material for: Gut development following insulin-like growth factor-1 supplementation to preterm pigs
Source: Pediatr Res. 2023 Dec 12;95(6):1528–35. doi: 10.1038/s41390-023-02949-9 (PMC11126387; doi:10.1038/s41390-023-02949-9)
Supplement: Supplementary file 1 — Supplementary Information [file 41390_2023_2949_MOESM1_ESM.pdf]

**Supplementary Table S1.** Parenteral and enteral supply

| Day | 1 | 2 | 3 | 4 | 5 | 6 | 7 | 8 | 9 |
|---|---|---|---|---|---|---|---|---|---|
| PN mL/kg/d | 96-120 | 96-120 | 144 | 96-144 | 72 | 72 | 72 | - | - |
| EN mL/kg/d | 32 | 48 | 64 | 72 | 96 | 112 | 128 | 128 | 144 |
| Day | 10 | 11 | 12 | 13 | 14 | 15 | 16 | 17 | 18 |
| PN mL/kg/d | - | - | - | - | - | - | - | - | - |
| EN mL/kg/d | 160 | 176-192 | 176-192 | 176-192 | 176-192 | 192-208 | 216-224 | 216-224 | 216-224 |

Volumes of parenteral and enteral nutrition for preterm pigs throughout the experiments. PN, parenteral nutrition. From day 11 in litter 1 and from day 1 in litter 2 and 3, PN and EN were adjusted to metabolic body weight by a factor calculated as weight ((g)/1000)0.7/(weight(g)/1000).

| Nutrient content | EN | PN, day 1-7 |
|---|---|---|
| Energy, kJ/L | 3872 | 3116 |
| Protein, g/L | 60 | 45 |
| Carbohydrate, g/L | 54 | 71 |
| Fat, g/L | 53 | 31 |

Enteral and parenteral diet composition for preterm pigs. The milk diet was based on raw, frozen bovine jersey milk, 7g/L vitamins (Phlexi-vit) and 25g/L of whey protein (DI9025, Arla Foods Ingredients) and 6 g/L of electrolytes (Revolyt Nutrition). PN, parenteral nutrition; EN, Enteral Nutrition.

Supplementary Table S2. Cause of death in pigs euthanized before the end of intervention

| Number | Litter | Postnatal age | Intervention | Cause of death |
|---|---|---|---|---|
| 1 | 1 | 4 | Control | NEC |
| 2 | 1 | 5 | Control | NEC |
| 3 | 1 | 8 | IGF-1 | NEC |

| | | | | |
|---|---|---|---|---|
| 4 | 1 | 9 | Control | NEC |
| 5 | 1 | 10 | IGF-1 | NEC |
| 6 | 1 | 11 | Control | Bowel obstruction |
| 7 | 1 | 17 | IGF-1 | Bowel obstruction |
| 8 | 1 | 18 | Control | Bowel obstruction |
| 9 | 2 | 3 | IGF-1 | NEC |
| 10 | 2 | 3 | Control | NEC |
| 11 | 2 | 3 | Control | NEC |
| 12 | 2 | 4 | IGF-1 | Iatrogenic: Perforation of catheter to abdomen. Subsequent parenteral nutrition fluid in the abdomen |
| 13 | 2 | 7 | Control | No obvious pathology |
| 14 | 2 | 11 | Control | Respiratory distress |
| 15 | 3 | 2 | IGF-1 | Respiratory distress |
| 16 | 3 | 3 | Control | NEC |

| | | | | |
|---|---|---|---|---|
| 17 | 3 | 3 | Control | NEC |
| 18 | 3 | 3 | Control | NEC |
| 19 | 3 | 3 | IGF-1 | Iatrogenic: Perforation of catheter to abdomen. Subsequent parenteral nutrition fluid in the abdomen |
| 20 | 3 | 4 | Control | NEC |
| 21 | 3 | 4 | Control | Respiratory distress |
| 22 | 3 | 4 | Control | Iatrogenic: Catheter perforation of umbilical artery intraperitoneally. Subsequent bleeding and shock |
| 23 | 3 | 4 | IGF-1 | NEC |
| 24 | 3 | 5 | IGF-1 | NEC |

| | | | | |
|---|---|---|---|---|
| 25 | 3 | 6 | Control | Iatrogenic: catheter related occlusion of vessel in hindleg with subsequent intractable pain. |
| 26 | 3 | 6 | Control | NEC |

## Supplementary Figure 1

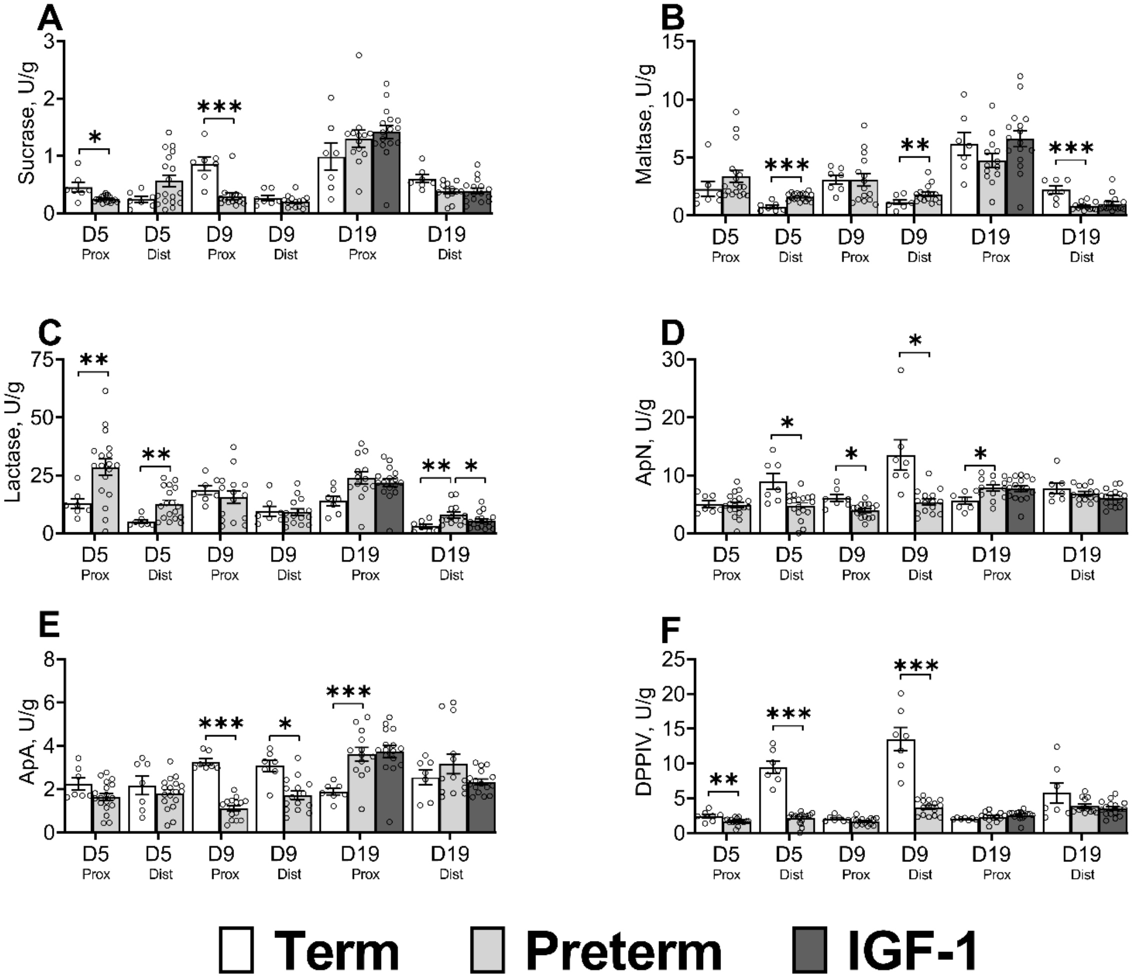


Supplementary figure 1:

Tissue-specific (U/g intestine) brush border enzyme activities in pigs born full-term (all n = 7), in control preterm pigs at postnatal ages 5 days (n = 18), 9 days (n = 15) or 19 days (n = 13), and in preterm pigs treated with rhIGF-1 until 19 days (IGF-1, n = 16). Comparisons between term and preterm control pigs and between preterm controls and preterm IGF-1 pigs was done separately. ApN, aminopeptidase N; ApA, aminopeptidase A; DPPIV, dipeptidylpeptidase; Prox, proximal small intestine; Dist, distal small intestine. Values are means ± SEM. *p < 0.05, **p < 0.01, ***p < 0.001.

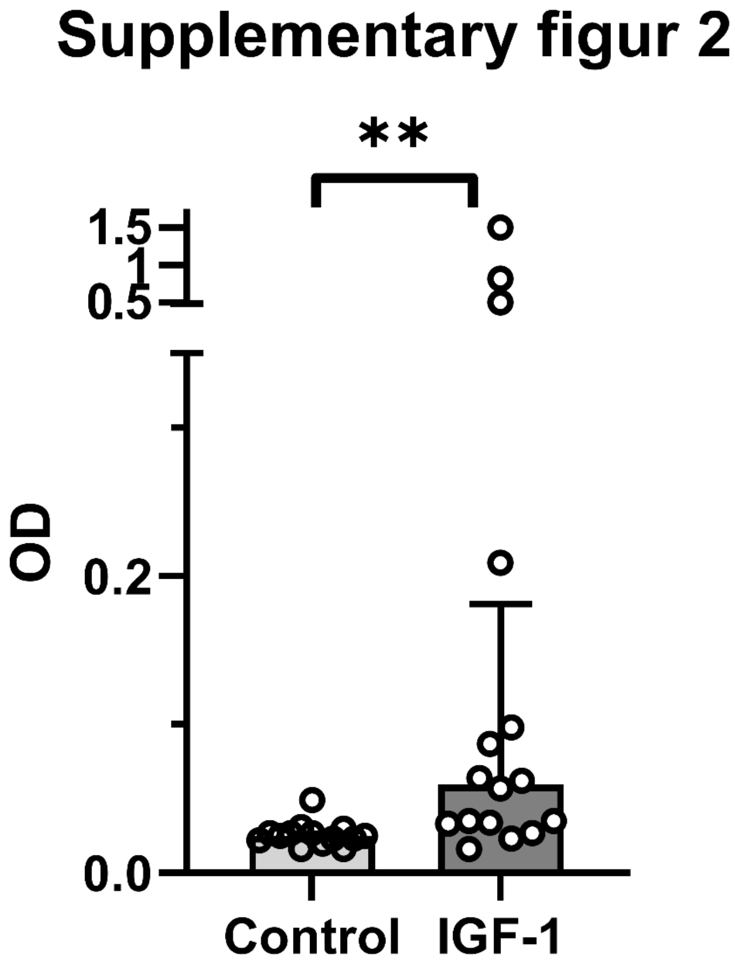


Supplementary Figure 2:

Levels of anti-rhIGF1/IGFBP3 antibody in pigs at postnatal day 19. Plasma anti-IGF-1/IGFBP3 antibody (IgG+IgA+IgM) was measured in control (n = 13) and IGF-1-treated pigs (n = 16) and represented as absorbance at 490 nm (OD). Values are median ± interquartile range, **$p < 0.01$.
